# miR-488-3p sponged by circ-0000495 and mediated upregulation of TROP2 in head and neck squamous cell carcinoma development

**DOI:** 10.7150/jca.40339

**Published:** 2020-03-05

**Authors:** Yanru Hao, Dejun Zhang, Yingyuan Guo, Zeming Fu, Duojiao Yu, Guofang Guan

**Affiliations:** Department of Otolaryngology, Head and Neck Surgery, The Second Hospital of Jilin University, Changchun 130041, P. R. China

**Keywords:** TROP2, prognostic marker, microRNA, circular RNA, head and neck squamous cell carcinoma

## Abstract

TROP2 (trophoblast cell surface antigen 2) overexpression has been reported in many human cancers. The correlation between TROP2 and tumor aggressiveness has implied it could be a prognostic indicator. However, the roles of TROP2 and their underlying mechanisms remain of great interest in head and neck squamous cell carcinoma (HNSCC) biology. In the current study, the prognostic significance of TROP2 in HNSCC archival samples was determined using immunohistochemistry. Quantitative reverse transcriptase PCR (qRT-PCR) was used to measure the phenotypic effects of TROP2 knockdown, miR-488-3p re-expression, and circRNAs expression. Cell viability, migration/invasion as well as *in vivo* tumor formation assays were accessed. The interactions of miRNAs-TROP2 or circRNAs-miRNAs were determined by qRT-PCR, western blot analysis and luciferase assays. TROP2 was demonstrated overexpression in HNSCC patients and cancer cell lines. High expression of TROP2 was significantly associated with patient relapse. TROP2 promoted tumor cell proliferation, migration, invasion, and tumor growth,* through AKT and MAPK pathways*. Further investigation revealed that TROP2 is a direct target of miR-488-3p, while circ-0000495 bounds to miR-488-3p. Our study unraveled a novel mechanism by which down-regulation of miR-488-3p sponged by circ-0000495 releases its epigenetic silencing to TROP2. The increased TROP2 promotes tumor proliferation, therefore, providing evidence in support of targeting the circ-0000495/miR-488-3p/TROP2 axis in contributing to HNSCC therapy and preventing tumor metastasis.

## Introduction

Head and neck squamous cell carcinoma (HNSCC) includes tumors from oral cavity, larynx, hypopharynx, and pharynx. It is the sixth most common malignancies worldwide 1. They are often diagnosed at advanced stages, leading to significantly high mortality and morbidity 2. Despite progress in combination with chemotherapy for more advanced stages, 30-40% of patients will still develop distant metastases within 5 years 3, 4, underlining the importance to better understand the molecular basis of tumor characteristics.

TROP2 (Trophoblast cell surface antigen 2), is also known as Tumor-Associated Calcium Signal Transducer 2 (TACSTD2), which belongs to a family with at least two closely related genes (TACSTD1- EpCAM, and TACSTD2- TROP2) 5. Overexpression of TROP2 has been clearly demonstrated in a wide range of human malignancies, including gastric 6, colorectal 7, cervical 8, oral cavity 9 and laryngeal carcinomas 10. Interestingly, the high expression of TROP2 has been noted to be associated with cancer progression and poor prognosis 5, 8, 9. We have previously characterized the prognostic value of TROP2 in a cohort of 58 Epstein-Barr virus (EBV) associated nasopharyngeal carcinoma (NPC) and demonstrated that TROP2 was an independent predictor for poor clinical outcome 11. However, there are controversial observations in regarding the regulation of TROP2 expression in cancer. TROP2 causes activation of the ERK1/3-MAPK pathway by increasing the levels of phosphorylated MAPK (ERK1/ERK2), which mediates cell cycle progression and protects cancer cells from apoptosis 12. TROP2 expression also significantly increased during gallbladder tumorigenesis through aberrant PI3K/AKT signaling 13. In contrast, a recent study demonstrated that TROP2 can be silenced by promoter methylation in the Tamoxifen-resistant breast cancer cell lines 14. Overall, this data emphasizes the need for investigation into causative mechanisms of TROP2 in carcinogenesis.

MicroRNAs (miRNAs) function by binding to mRNA transcripts with complementary sequences and inhibit their expression 15. Most notably, they have been found to dysregulate in human malignancies and affect cancer prognostic, predictive, and therapeutic avenues through various mechanisms 15, 16. miRNAs act either as promotors (oncogenes) or suppressors of tumorigenesis 17. Numerous miRNAs have been reported to be dysregulated in head and neck squamous cell carcinoma. miR-let-7a, miR-34c-5p, miR-21, miR-196b, and miR-375 have been shown association with laryngeal carcinoma progression 18-20; while miR-155, miR-185 were predictors for survival in oral cavity squamous cell carcinoma 21. A set of miRNAs were identified to be associated with HPV status in oropharyngeal carcinoma and correlated with clinical outcome 22. Therefore, investigation on whether microRNAs correlate or mediate TROP2 expression may shed light for novel therapeutic strategies.

Recently, a subclass of novel non-coding RNAs-circular RNAs (circRNAs) has attracted much attention. circRNAs are abundant endogenous non- coding RNA with no 5′ cap and 3′ polyadenylation tail and are much more stable than their linear counterparts 23. circRNAs can act as competitive endogenous RNAs to sponge miRNAs and regulate their transcription 24. Studies have shown that circRNAs are key regulators of various diseases and are involved in tumorigenesis and invasion. However, the function of most circRNAs remains largely unknown.

In the current study, we reported that the overexpression of TROP2 in HSNCC, and the higher TROP2 expression was associated with worse clinical outcome. Further investigations revealed that the dysregulated TROP2 was associated with enhanced tumor progression both *in vitro* and *in vivo,* through AKT and MAPK pathways. Moreover, our study unraveled a novel mechanism by which down- regulation of miR-488-3p sponging by circ-0000495 releases its epigenetic silencing to TROP2, provide evidence in support of targeting the circ-0000495/ miR-488-3p/TROP2 axis in contributing to HNSCC therapy and preventing metastasis.

## Materials and Methods

### Cell lines and patient information

The three human head and neck cancer cell lines: FaDu (human hypopharyngeal squamous cancer), SCC-22 (hypopharyngeal carcinoma), SCC-9 (human tongue squamous cell carcinoma) obtained from the American Type Culture Collection, and NOE cells (normal oral epithelial cells), from Celprogen (San Pedro, CA) were maintained in DMEM with 10% fetal bovine serum (FBS). All cells were maintained in a 37 °C incubator with a humidified 5% of CO2 and tested to be free from mycoplasma contamination.

With approval from the Research Ethics Board at the Second Hospital of Jilin University, forty-two diagnostic formalin-fixed paraffin-embedded (FFPE) blocks were collected from 24 laryngeal and 18 hypopharyngeal carcinoma patients between 2003 and 2015. All studied tumor specimens were assessed on hematoxylin-and-eosin-stained slides using standard diagnostic criteria. Clinical characteristics of these cancer patients are provided in Table 1.

### Immunohistochemistry

TROP2 expression was determined by immunohistochemistry staining using the standard immunoperoxidase staining procedure. A purified goat polyclonal antibody (AF650, 1/50 dilution, R&D Systems), which identifies TROP2 extracellular domain was used as described previously 11. The Universal LSAB^TM^2 detective system (DakoCytomation) was used. Ki-67 expression was evaluated by using the monoclonal antibody (DakoCytomation). Sections without primary antibodies were used as negative controls. Positive TROP2 expression was assessed by calculating the tumor cell membrane staining proportion and intensity. The overall score was a combination of staining proportion and intensity as described previously 11: 0=none; 1<10%; 2=10-50%; 3≥50%. Immunostaining of Ki-67 was evaluated by counting at least 500 tumor cells in the five most densely staining fields at high power (x400). The percentage of positive staining tumor nuclei was calculated.

### Quantification of TROP2, miRNAs and circRNAs

Total RNA was extracted from three cell lines using the RNA extraction kit from Qiagen according to the manufacturer's instructions. The RNA was reversely transcribed using SuperScript II Reverse Transcriptase (Invitrogen) according to the manufacturer's recommendations. Quantitative real-time PCR analysis was performed using SYBR Green PCR Master Mix (Applied Biosystems). GAPDH was used as endogenous control. The expression of hsa- miR-15a-3p, has-miR-448-3p, and has-miR-550b-2-5p were measured using the standard Taqman MicroRNA assay (Applied Biosystems). RNU48 were used as endogenous controls. For circRNAs, total RNA was treated with RNase R, genomic DNA was isolated using QIAamp DNA Mini kit (Qiagen). SYBR green™Premix Ex Taq™ kit (TaKaRa, Dalian, China) was used for real-time PCR (qRT-PCR). 18S rRNA was utilized as internal control. qRT-PCR was performed in an ABI 7900 real-time PCR system (Applied Biosystems). Primers for qRT-PCR are provided at supplementary Table 1. The relative expression of RNAs was calculated by 2-ΔΔCt method.

### Cell transfection and proliferation assays

The biological effects of TROP2 were investigated by transfection of TROP2-target siRNA (siTROP2) (Tagman Silencer select siRNA, Thermofisher) using the LipofectAMINE 2000 (Invitrogen) reverse transfection protocol. All cells were transfected at a final concentration of 40 nM. The cytopathic effects of Fadu and SCC-9 cells transfected with siTROP2 were evaluated using the CellTiter 96 Non-Radioactive Cell Proliferation Assay (MTS) (Promega Bio Sciences). Cell proliferative activity was measured at 24, 48 and 72 hours after transfection.

### Cell migration and invasion

The cellular effects of knockdown TROP2 were further investigated in FaDu and SCC-9 cells for cell migration and invasion using the BD Biosciences BioCoat control chamber and Matrigel invasion chamber. 1x10^5^ cells were transfected with siTROP2 or siRNA scramble control, and plated on either the 6-well control inserts (PET membrane) or trans-well chambers pre-coated with Matrigel. A medium containing 15% fetal bovine serum in the lower chamber served as the chemo-attractant. After 24 hours' incubation, non-migrating or invading cells were removed from the upper surface of the membrane with cotton swabs. The migrating or invasive cells attached to the low surface of the membrane insert were then fixed and stained with Diff-Quick Stain (BD Biosciences). The number of migrating or invasive cells was counted under a microscope.

### Western blotting

Fadu cells were transfected with either siTROP2 or scramble control (SC). After 72 hours post-transfection, the cells were collected and lysed. Protein extracts were prepared and quantified using the BCA method. 20 µg of protein were loaded onto 10% Tris-glycine protein gels, and then transferred onto a nitrocellulose membrane. Membranes were blocked in 5% milk in Tris-buffered saline with 0.1% Tween-20 (TBST). The membranes were probed with mouse anti TROP2 (R&D systems), p-p44/42, Erk1/2, p-AKT, AKT, AP-1(c-Jun) (Cell signaling technology), and GAPDH (Abcam, USA) antibodies for overnight and was followed by incubation with the second antibodies (Abcam, USA) labeled with horseradish peroxidase for 2 hours. Signals were visualized using the ECL western blotting substrate system.

### Luciferase assay

To assess the regulations between circRNAs with miRNAs or miRNAs with TROP2, Dual-report luciferase assays were used. The sequences of circ-0000495 or TROP2 3'-UTR regions, which contained wild-type or mutant miR-488-3p putative binding sites were synthesized respectively. The products were then inserted into pMIRREPORT luciferase vectors (Ambion). The direct interaction between 3'UTR of TROP2 or circ-0000495 with miR-488-3p was detected by co-transfection of the wild-type or mutated vector with premiR-488-3p or scramble control in Fadu and SCC-9 cell lines. pRL-SV40 vector (Promega) containing Renilla luciferase was also transfected to each well. At 48 hours post-transfection, cells were harvested and Dual-Glo luciferase assay system (Promega) was used to assess both firefly and Renilla luciferase activities.

### *In vivo* experiments

For tumor formation assay, 6‐ to 8‐week‐old severe combined immunodeficient (SCID) BALB/c female mice were purchased from Jilin University Experimental Animal Center and conducted experiments in compliance with the Jilin University Experimental Animal Ethical Inspection. Fadu cells were transfected with siTROP2, SC or LipofectAMINE 2000. At 48 hours post‐transfection, all cells were harvested, 5 × 10^5^ cells from each group were mixed with an equivalent volume of medium, and injected subcutaneously onto the back of SCID mice. Tumor volume was measured using a vernier caliper twice weekly and calculated using the following equation: (tumor length × width^2^)/2 25.

### Statistical analysis

Box plots were utilized to visually explore the expression of TROP2, miRNAs or circRNAs. GraphPad Prism (version 6.0) was utilized to perform the statistical analysis. All data were expressed as the mean ± SE; a p-value of <0.05 was considered to be statistically significant.

Overall survival (OS) was defined from the time of diagnosis to date of any deaths or last follow-up. Disease-free survival (DFS) was defined from the time of diagnosis to the date of first failure or last follow-up. Survival curves were generated using the Kaplan-Meier method and compared by means of the log-rank test. Associations between TROP2 expression and clinicopathological variables were assessed with the Chi-test. In the univariate model, if factors with prognostic significance, they were then further analysed in a multivariate Cox's proportional hazards regression model. A p-value of less than 0.05 was considered to be statistically significant.

## Results

### TROP2 overexpressed in HNSCC and associated with tumor recurrence

**Figure 1:**
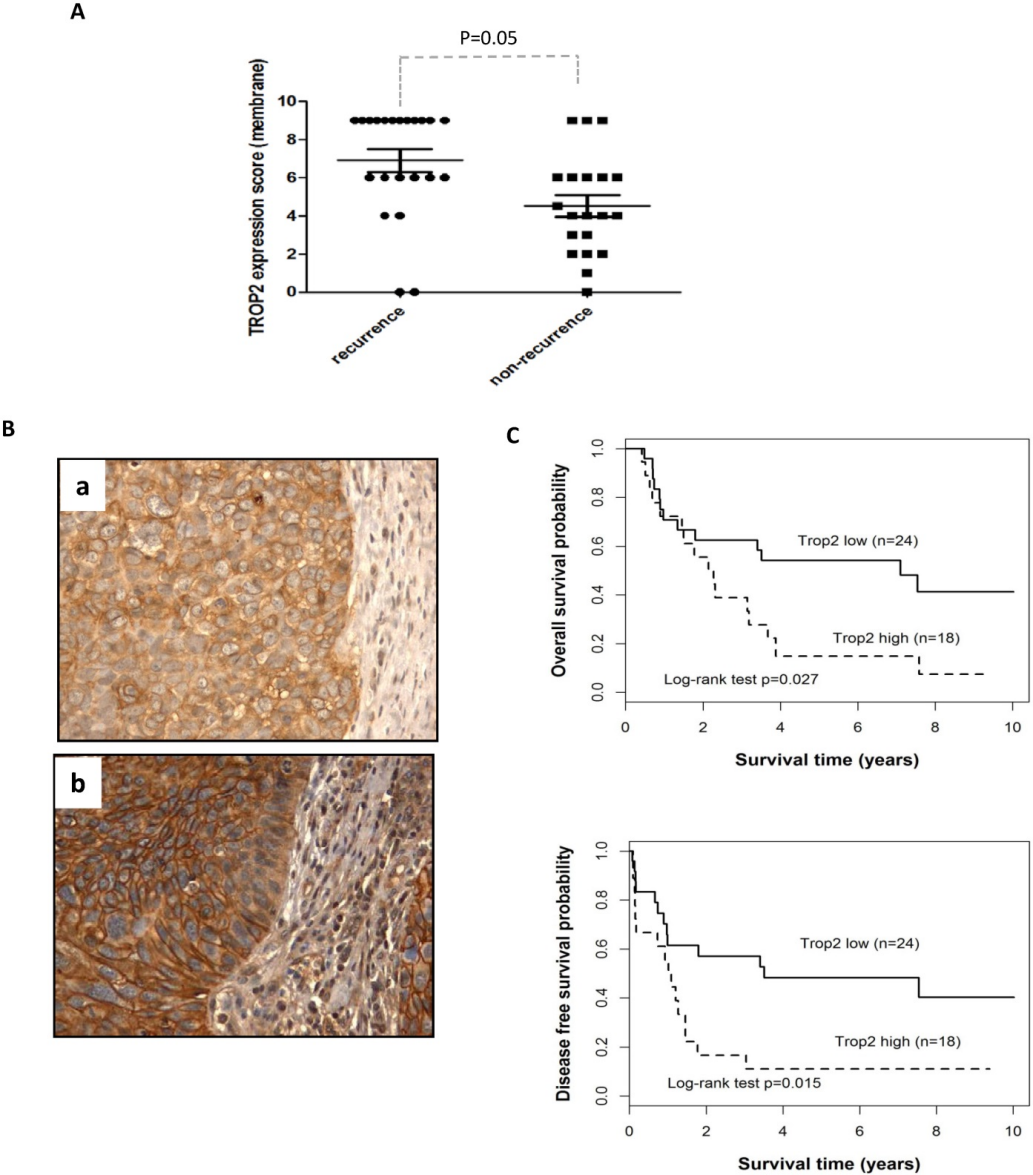
** Overexpression of TROP2 is associated with poor survival of patients with HNSCC. (A)** Immunohistochemistry analysis was used to evaluate TROP2 expression in HNSCC patient samples, demonstrating that TROP2 was significantly upregulated in tumor tissue and associated with tumor recurrence (p=0.05). **(B)** Representative images showing a weak (a) and a strong (b) tumor cell membrane staining for TROP2. **(C)** Kaplan-Meier survival curves showed a significant difference in both overall survival (p=0.027) and disease-free survival (p=0.015) when comparing high vs. low expression groups.

To validate the clinical importance of TROP2 in HNSCC, the gene expression was evaluated in 42 HNSCC patients, including 24 laryngeal and 18 hypopharyngeal carcinomas. The majority of cancer cells showed a homogeneous moderate to strong membranous expression of TROP2 compared with adjacent normal epithelial cells (showed no or weak staining). TROP2 was exhibited a higher expression level in recurrence than those in non-recurrence patients (Figure 1A, p=0.05). Figure 1B presented weak (a) and strong (b) membranous staining of TROP2. Furthermore, Kaplan-Meier survival analysis showed that patients with higher TROP2 expression level associated with worse overall survival (OS) (Figure 1C, top, p=0.027; Table 2 A), as well as with worse disease-free survival (DFS) (Figure 1C, bottom, p=0.015; Table 2 A), compared with those with lower TROP2 expression. However, TROP2 expression was not significantly correlated with gender, age, tumor stage or tumor site (all p>0.1; Table 2 A) by univariate analysis. Multivariate analysis also suggested that TROP2 overexpression was an independent prognostic factor for both OS and DFS (p=0.031 and p=0.018, respectively; Table 2 B).

### Effects of TROP2 on cell proliferation, migration, and invasion

**Figure 2:**
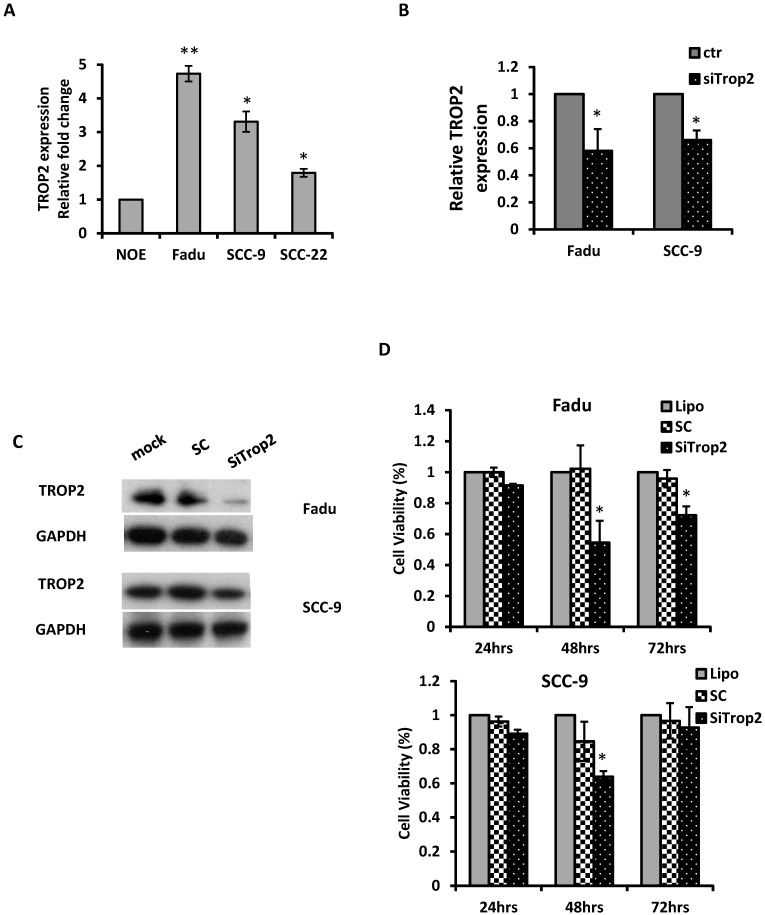
** Knockdown of TROP2 significantly reduces cell proliferation. (A)** qRT-PCR analysis demonstrated that TROP2 was up-regulated in 3 HNSCC cell lines. **(B)** siRNA for TROP2 reduced TROP2 expression in both Fadu and SCC-9 cells. *, p<0.05. **(C)** Western blot showed a reduction of TROP2 levels in both Fadu and SCC-9 cell lines at 72 hours post-transfection. SC: scramble control. **(D)** siRNA for TROP2 was transfected into Fadu or SCC-9 cells and significantly reduced cell proliferation, particularly at 48 hours post-transfection, compared to scramble control, using the MTS assay. *, p<0.05.

**Figure 3:**
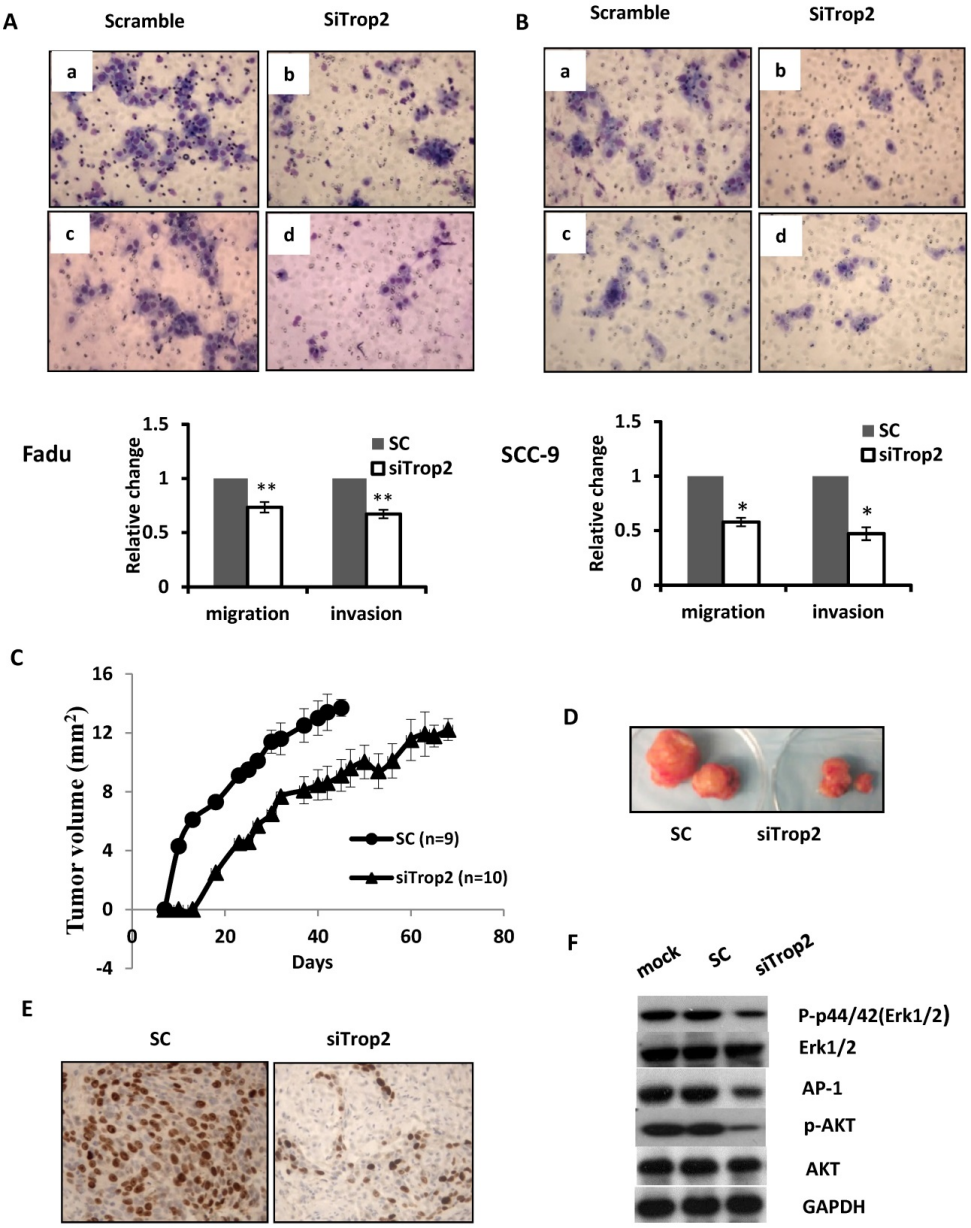
** Depletion of TROP2 regulates cell migration, invasive and suppresses tumor growth through AKT and MAPK pathways. (A and B)** Representative images and quantification depicting the reduction of migratory ability (top) and invasion (bottom) of Fadu and SCC-9 cells that were transfected with 40uM of siTROP2 compared to negative scramble control. All data represent the mean ± SE from 3 independent experiments. *, p<0.05; **, p<0.01. **(C and D)** The Fadu cells were transfected with siTROP2 or negative scramble control. 48 hours after, the harvested cells were injected into mice subcutaneously. The tumor formation was then monitored twice a week. The indicated tumor volume represents the mean ± SE, (*, p<0.05). **(E)** Representative photomicrographs of Ki-67 immunostaining in Fadu xenograft tumors; quantitation of the number of proliferation cells in 5 representative hpf's. **(F)** Western blotting for p-Erk1/2, total Erk1/2, AP-1, p-AKT, and total AKT at 72 hours post-transfection of siTROP2 in Fadu cells. SC: scramble control; hpf's: high power fields.

The biological significance of TROP2 was assessed in cancer cells. The expression of TROP2 was evaluated in 3 HNSCC cancer cell lines: Fadu (hypopharyngeal carcinoma), SCC-9 (tongue carcinoma), and SCC-22 (hypopharyngeal carcinoma) compared with that of the normal oral epithelial cell line NOE. TROP2 was significantly upregulated in all cancer cell lines (Figure 2A). siRNA specifically against TROP2 was then transfected into Fadu or SCC-9 cells. Of note, the TROP2 expression was significantly reduced in both cells, by 38% and 24%, respectively (Figure 2B). Those reductions were further confirmed by western blot analysis (Figure 2C). Furthermore, the reductions in TROP2 expression led to significant decreases in cell viability for both cell lines, particularly at 48 hours post-transfection compared to that in the control (Figure 2D). In addition, knockdown of TROP2 resulted in a significant reduction in cell migration (48%) and invasion (55%) of Fadu cells compared to that of the scramble control (Figure 3A). A similar reduction in cell migration and invasion was also observed in SCC-9 cells (Figure 3B).

### Knockdown of TROP2 suppressed tumor growth *in vivo*

Given the strong association between TROP2 overexpression and patient relapse, we seek whether TROP2 knockdown could affect tumor growth *in vivo*. Fadu cells were treated either with siTROP2 or scramble siRNA control (SC) and collected 48 hours post-transfection. The same number of cells from each group was then injected into BALB/c nude mice subcutaneously. TROP2 knockdown cells showed significantly delayed tumor growth compared with mice injected with SC (p=0.047, Figure 3C & D). To acquire further insights into the mediators of suppression of TROP2 leading to reduced tumor growth, tumors from the SC and TROP2 knockdown groups were collected. Tumor proliferation analysis showed a significant reduction of Ki-67 expression in the TROP2 depletion group (p<0.01, Figure 3E).

### TROP2 induced tumor growth through different pathways in HNSCC

PI3K/AKT pathway has been known to be involved in cell proliferation and invasion in a variety of cancer 26, 27. It has been also shown that the mitogen-activated protein kinase (MAPK) pathway can be activated and linked to cell differentiation and apoptosis, and also has been associated with survival 28. To analyze the underlying mechanisms conferred by TROP2 on HNSCC cells, Fadu cells were treated with lipofectimine 2000 alone, SC or siTROP2 (40uM), the cell pellets were then harvested for western blot analysis at 72 hours. As shown in Figure 3F, knockdown of TROP2 decreased the phosphorylated levels of AKT, p44/42(Erk1/2) and AP-1, while the levels of AKT and Erk1/2 did not change.

### miR-488-3p directly targeted TROP2

**Figure 4:**
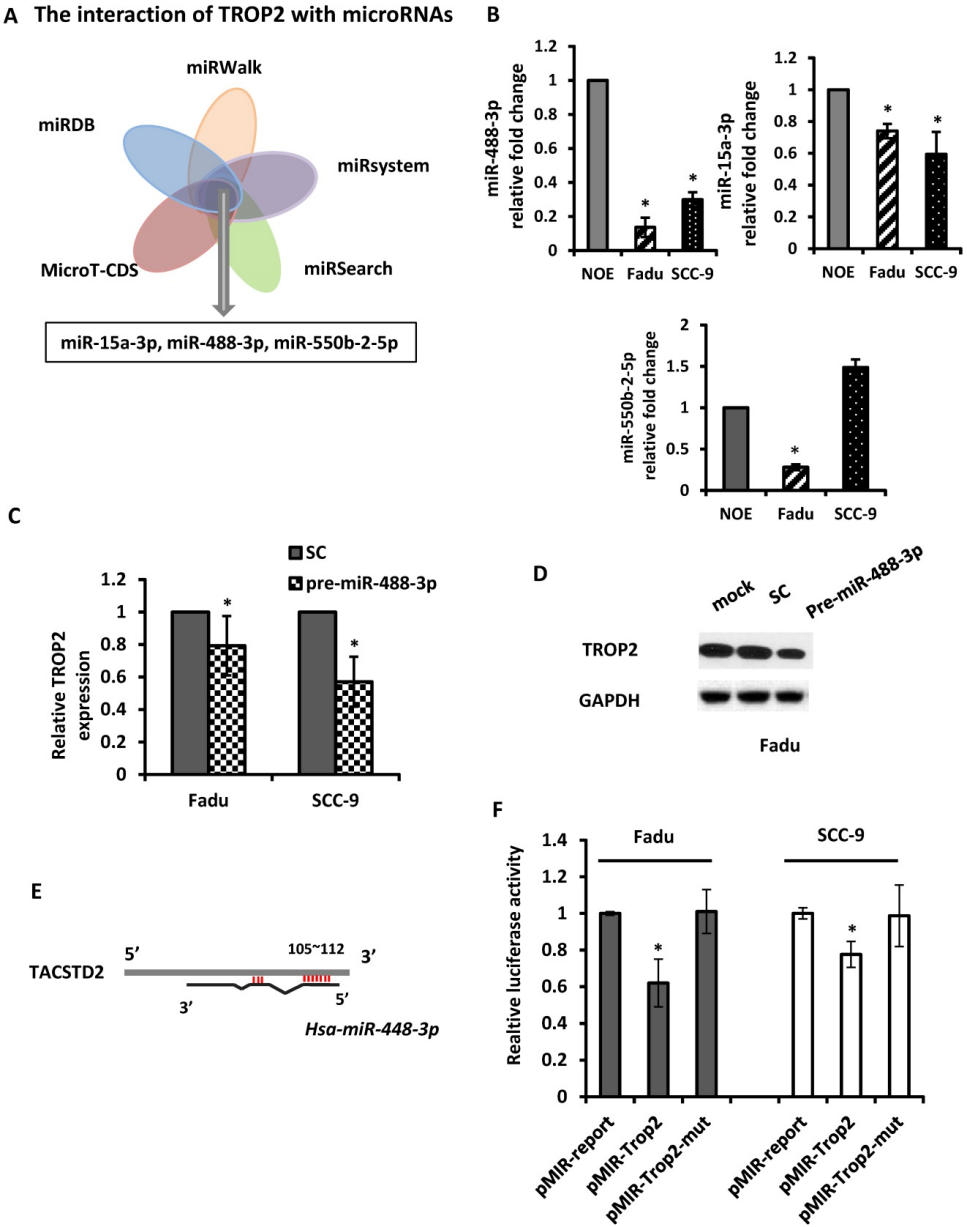
** miR-488-3p directly targets TROP2 in HNSCC. (A)** A combinatorial approach of five *in silico* microarray datasets and Kyoto Encyclopedia of Genes and Genomes (KEGG) Pathway and Gene Ontology (GO) enrichment analyses were performed. **(B)** Baseline expression of putative miRNAs (miR-15a-3p, miR-488-3p, and miR-550b-2-5p) analyzed by qRT-PCR. *, p<0.05. **(C and D)** qRT-PCR and Western blotting analyzed the effects of TROP2 expression after increasing miR-488-3p expression by mimic's miR. *, p<0.05. **(E)** A predicted miR-488-3p target site on TACSTD2 (TROP2) 3'-UTR was illustrated. miRNA seed sequence was shown in red. **(F)** Luciferase reporter assays of Fadu and SCC-9 cells co-transfected with pMIR-Trop2 or pMIR-Trop2-mut plasmids, and either premiR-488-3p or scramble negative control miR. Samples were analyzed 72 hours post-transfection and data normalized to the pMIR report only transfection. Data represents the mean ± SE, n = 3. *, p< 0.05. SC: scramble control.

The mechanism of upregulation of TROP2 in HNSCC remains unclear. As microRNAs play a key role in regulating their downstream genes and have effects on physiological function. Here, we utilized 5 *in silico* gene target-prediction softwares- miRDB, miRWalk, miRSystem, MicroT-CDS and miRSearch to predict microRNAs, which may potentially target to TROP2. Based on their various biological effects on cellular processes and/or tumor progression, three potential tumor suppressor miRNAs were selected for further study: miR-15a-3p, miR-488-3p and miR-550b-2-5p (Figure 4A). The baseline expression of each miRNAs was examined by individual qRT-PCR assays. The results demonstrated that miR-488-3p and miR-15a-3p were indeed under-expressed in both Fadu and SCC-9 cells comparing to that in NOE cells (Figure 4B). Mimics of miR-488-3p resulted in down-regulation of TROP2 in both Fadu and SCC-9 cells (Figure 4C & D), while the level of TROP2 did not change significantly by introducing mimics of miR-15a-3p (data not shown). These findings indicated that miR-488-3p is highly probable to target TROP2 in HNSCC cells.

In order to establish a direct interaction between miR-488-3p and the 3'-UTR of TROP2, we constructed several reporter vectors carrying the predicted binding site(s) downstream of a firefly luciferase gene in the pMIR-Report vector as previously described 29. Then, a luciferase assay was employed. A luciferase constructed plasmid was designed and made for TROP2. The overlapping seed site of miR-488-3p with TROP2 was illustrated in Figure 4E. Either Fadu or SCC-9 cells were co-transfected with premiR-488-3p (or a scramble control) and pmiR-TROP2-3' UTR (or a mutated pmiR-TROP2-3' UTR). The luciferase activity was measured and compared with that in cells co-transfected with NC. The luciferase reporter that contained the TROP2 3' UTR was significantly decreased by premiR-488-3p, whereas the mutated reporter was not affected (Figure 4F).

### Circ-0000495 interacted TROP2 by sponging miR-488-3p

**Figure 5:**
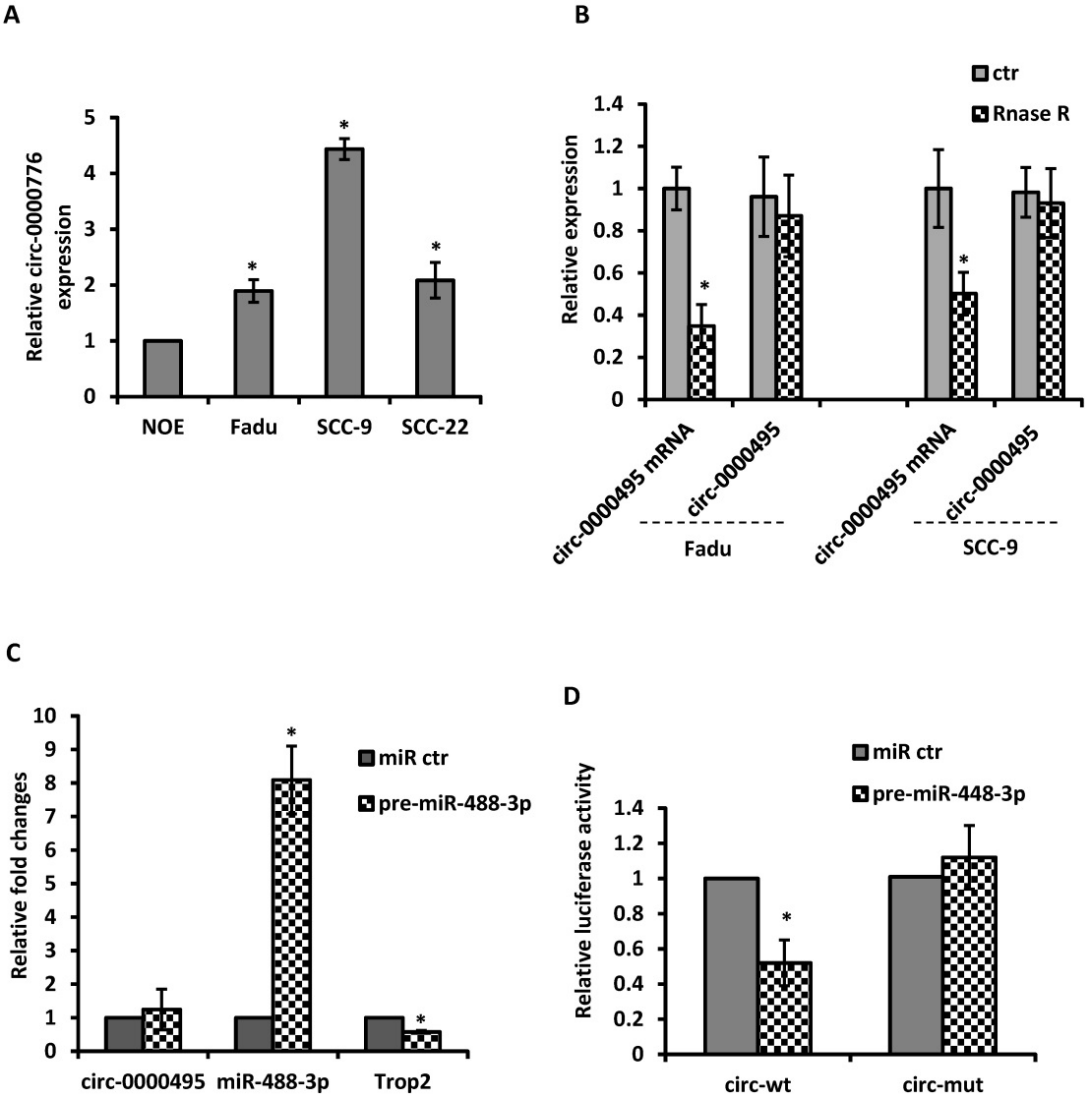
** circ-0000495 sponges miR-488-3p in HNSCC cells. (A)** qRT‐PCR assay suggested the expression of circ‐0000495 was upregulated in HNSCC cell lines. **(B)** qRT‐PCR analysis of the expression of circ‐0000495 after RNase R treatment, indicated the stability of circ‐0000495 than that of circ‐0000495 mRNA. *, p< 0.05. **(C)** Mimics of miR-488-3p significantly reduced TROP2 expression but no effects on circ-0000495 expression. **(D)** Luciferase activity in SCC-9 cells co‐transfected with luciferase reporter containing circ‐0000495 sequences with wild‐type or mutated miR‐488-3p binding sites and the mimics of miR‐488-3p or control. Data was represented as means ± SE, n=3. * p< 0.05.

Given the evidence that circRNAs can modulate miRNA activity, we then explore the potential interaction between circRNAs with miR-488-3p. By utilizing circular RNA interactome (https://circinteractome.nia.nih.gov/) and TargetscanHuman 7.2 (http://www.targetscan.org/vert_72/), the top 5 up-regulated circRNAs were selected based on their higher context score percentile and the number of binding sites. Through the functional analysis with KEGG pathway of HNSCC, circ-0000495 was selected as a candidate circRNA to investigate. The qRT‐PCR analysis showed that circ-0000495 has higher expression in Fadu and SCC-9 cells than that in normal control cells (Figure 5A). After RNase R treatment, the linear RNAs were significantly reduced, but the circular RNAs were more stable and resisted to RNase R (Figure 5B). When pre-miR-488-3p was transfected to Fadu cells, the miR-488-3p level was significantly increased, while TROP2 expression decreased significantly (Figure 5C). However, circ-0000495 level had no significantly change (Figure 5C). To assess whether circ‐0000495 acts as an antagonist/sponge for miR-488-3p, the dual‐luciferase reporter assay was performed. The reduced luciferase activity in circ-0000495 after transfected with mimics of miR-488-3p (wild type) indicated that circ-0000495 could directly bind to miR-488-3p (Figure 5D). The binding sites between circ‐0000495 with miR-488-3p are shown in supplementary Figure 1.

## Discussion

TROP2 is overexpressed in many cancers and has been associated with disease progression and recurrence 12, 30. In the current study, we provide evidence that TROP2 significantly overexpresses in primary HNSCC samples as well as cancer cell lines. The overexpression of TROP2 promotes tumor cell proliferation, migration, and invasion *in vitro* and *in vivo* by regulating AKT and ERK/MAPK signaling pathways. Knockdown of TROP2 in Fadu and SCC-9 cells presents inhibitory effects on the malignant behaviors. Moreover, aberrant expression of TROP2 significantly correlates with poor overall survival and disease-free survival, suggesting that TROP2 can be considered an independent prognostic predictor for HNSCC patients.

Although increasing evidence in the literature documents overexpression of TROP2 in human malignancies, the precise mechanism leading to TROP2 overexpression in the development and progression of cancer remains largely unknown. Only few pathways have been described which can lead to TROP2 gene deregulation. TROP2 can affect signaling by interaction with neuregulin 1, resulting in inhibition of ErbB3 (HER3) in head and neck squamous cell cancer 31. It is also involved in calcium signaling by binding PIP2 and phosphorylating by PKC 32. The phosphorylation of TROP2 could, in turn, activate the Raf and NF-κB pathways 33. In addition, the activation of ERK1/2-MAPK pathways by TROP2 contributes to cell progression 12. Our data are in line with above observations and further confirm the roles of AKT and ERK/MAPK signaling pathways in regulating TROP2 expression in HNSCC. According to previous studies, tumor stage is an important prognostic factor for overall survival of head and neck cancers 34, 35, however, in our study, the prognostic values of T category for both OS and DFS did not show the significant differences. It is possible that the difference may be due to our relative small sample size, and clearly, further studies are required to make this observation more robust.

In recent years, microRNAs are increasingly recognized as a major class of regulatory molecules, in which they mediate tumor development and progression, primarily through the regulation of their respective target genes for degradation and inhibiting protein translation 36. Using in silico bioinformatics tools and biochemical validations, we demonstrated that miR-488-3p, which is under-expressed in HNSCC, could negatively regulate TROP2 expression. miR-488-3p has been reported as a tumor suppressor in several cancers. It has been shown a reduction level in small intestinal neuroendocrine tumors by microRNA profiling 37. The downregulation of miR-488-3p was also associated with advanced TNM stage in gastric cancer 38. Our findings support with only one previous study, in which TROP2 is the direct target of miR-125b-1 and loss of miR-125b-1 by hypermethylation releases the inhibition to TROP2 39. We provide evidence for an alternate mechanism for TROP2 overexpression through miR-488-3p in HNSCC.

The novelty of our current study, however, is the link to the circRNAs with the regulation of TROP2. Although functions of most circRNAs remain elusive, some circRNAs are shown to be functional in gene expression regulation and potentially relate to diseases 40. One major function of circRNAs is acting as competing endogenous RNAs (ceRNAs) that could sponge miRNAs to regulate mRNAs expression 41. The mechanism for miR-488-3p down-regulation are complex, recent studies have provided new biological explanation for the regulation of miRNAs, in which demonstrated circ‐SERPINE2 can promote cell proliferation by sponging miR‐375 and regulating YWHAZ expression in gastric carcinoma cells 42. The other group has also observed that circVAPA is up-regulated in colorectal cancer and promote tumor progression by sponging miR-101 43. Using bioinformatics analysis and biochemistry tools, we demonstrated circ-0000495 has putative binding sites with miR-488-3p. The luciferase assays indicated that there is a direct negative interaction between circ-0000495 and miR-488-3p. Although the function of circ-0000495 is not clear, the best transcript of circ-0000495 has been identified as MYCBP2 (http://www.circbase.org/cgi-bin/singlerecord.cgi?id=hsa_circ_0000495), which encodes an E3 ubiquitin-protein ligase and regulates mTOR signaling 44. Our current data further supports above observations and have unraveled another mechanism by which circ-0000495 could promote tumor progression through modulating TROP2 expression by sponging miR-488-3p in HNSCC cells. To the best of our knowledge, this is the first report describing the function of circ-0000495 and linking the regulation of TROP2 by the circ-0000495 /miR-488-3p axis to head neck cancer progression.

In conclusion, a pleiotropic oncogenic role for TROP2 has been identified in HNSCC, in which its over-expression promotes tumor progression and associates with patient survival by regulating AKT and ERK/MAPK signaling pathways. Furthermore, a novel mechanism has been demonstrated, by which up-regulated circ-0000495 mediates overexpression of TROP2 by sponging miR-488-3p. Hence, this axis of circ-0000495/miR-488-3p/TROP2 might shed new light for the potential therapeutic strategy for HNSCC.

## Supplementary Material

Supplementary figure and table.

## Figures and Tables

**Table 1:** Patient characteristics (n=42)

Variables		No. of patients (%)
**Age (range)**		65.8 (50.4-87.6)
**Gender**		
	Female	6(14%)
	Male	36(86%)
**Tumor stage**		
	II	4(10%)
	III-IV	38(90%)
**Tumor site**		
	Larynx	24(57%)
	Hypopharynx	18(43%)
**TNM**		
	T1-T2	10(24%)
	T3-T4	32(76%)
**Relapse**		
	Yes	21(50%)
	No	21(50%)

**Table 2:** Univariate and multivariate analyses of prognostic parameters.

**A.** Univariate analyses					
**Parameter**	**OS**			**DFS**	
	***p* value**	**HR(95%CI)**		***p* value**	**HR(95%CI)**
**Sex** (Male / Female)	0.119	2.971(0.705-12.521)		0.131	2.89(0.683-12.224)
**Age** (<=65 / >65)	0.153	0.585(0.278-1.231)		0.171	0.598(0.284-1.259)
**T stage** (TI-II / TIII-IV)	0.579	1.291(0.523-3.183)		0.449	1.412(0.573-3.478)
**Tumor site** (Larynx/ Hypopharynx)	0.107	0.547(0.26-1.151)		0.212	0.629(0.302-1.313)
**TROP2 overexpression** (H/L)	0.027	2.265(1.076-4.767)		0.015	2.473(1.164-5.257)
**B.** Multivariate analyses					
**Parameter**	**OS**			**DFS**	
	***p* value**	**HR(95%CI)**		***p* value**	**HR(95%CI)**
**T stage** (TI-II / TIII-IV)	0.571	1.290(0.523-3.183)		0.438	1.412(0.573-3.478)
**Tumor site** (Larynx/ Hypopharynx)	0.115	0.546(0.260-1.150)		0.217	0.629(0.302-1.312)
**TROP2 overexpression** (H/L)	0.031	2.265(1.076-4.767)		0.018	2.47(1.164-5.257)

H: high expression; L: low expression

## References

[1] Siegel R, Naishadham D, Jemal A (2012). Cancer statistics, 2012. CA Cancer J Clin.

[2] Kim L, King T, Agulnik M (2010). Head and neck cancer: changing epidemiology and public health implications. Oncology (Williston Park).

[3] Leemans CR, Braakhuis BJ, Brakenhoff RH (2011). The molecular biology of head and neck cancer. Nat Rev Cancer.

[4] Gregory CD, Kirchgens C, Edwards CF, Young LS, Rowe M, Forster A (1987). Epstein-Barr virus-transformed human precursor B cell lines: altered growth phenotype of lines with germ-line or rearranged but nonexpressed heavy chain genes. European journal of immunology.

[5] Fong D, Moser P, Krammel C, Gostner JM, Margreiter R, Mitterer M (2008). High expression of TROP2 correlates with poor prognosis in pancreatic cancer. Br J Cancer.

[6] Muhlmann G, Spizzo G, Gostner J, Zitt M, Maier H, Moser P (2009). TROP2 expression as prognostic marker for gastric carcinoma. J Clin Pathol.

[7] Ohmachi T, Tanaka F, Mimori K, Inoue H, Yanaga K, Mori M (2006). Clinical significance of TROP2 expression in colorectal cancer. Clin Cancer Res.

[8] Liu T, Liu Y, Bao X, Tian J, Liu Y, Yang X (2013). Overexpression of TROP2 predicts poor prognosis of patients with cervical cancer and promotes the proliferation and invasion of cervical cancer cells by regulating ERK signaling pathway. PLoS One.

[9] Fong D, Spizzo G, Gostner JM, Gastl G, Moser P, Krammel C (2008). TROP2: a novel prognostic marker in squamous cell carcinoma of the oral cavity. Mod Pathol.

[10] Wu H, Xu H, Zhang S, Wang X, Zhu H, Zhang H (2013). Potential therapeutic target and independent prognostic marker of TROP2 in laryngeal squamous cell carcinoma. Head Neck.

[11] Guan GF, Zhang DJ, Wen LJ, Yu DJ, Zhao Y, Zhu L (2015). Prognostic value of TROP2 in human nasopharyngeal carcinoma. Int J Clin Exp Pathol.

[12] Shvartsur A, Bonavida B (2015). Trop2 and its overexpression in cancers: regulation and clinical/therapeutic implications. Genes Cancer.

[13] Li X, Teng S, Zhang Y, Zhang W, Zhang X, Xu K (2017). TROP2 promotes proliferation, migration and metastasis of gallbladder cancer cells by regulating PI3K/AKT pathway and inducing EMT. Oncotarget.

[14] Zimmers SM, Browne EP, Williams KE, Jawale RM, Otis CN, Schneider SS (2018). TROP2 methylation and expression in tamoxifen-resistant breast cancer. Cancer Cell Int.

[15] Croce CM (2009). Causes and consequences of microRNA dysregulation in cancer. Nat Rev Genet.

[16] Bartel DP (2004). MicroRNAs: genomics, biogenesis, mechanism, and function. Cell.

[17] Calin GA, Croce CM (2006). MicroRNA signatures in human cancers. Nat Rev Cancer.

[18] Hu A, Huang JJ, Xu WH, Jin XJ, Li JP, Tang YJ (2015). MiR-21/miR-375 ratio is an independent prognostic factor in patients with laryngeal squamous cell carcinoma. Am J Cancer Res.

[19] Zhao X, Zhang W, Ji W (2018). miR-196b is a prognostic factor of human laryngeal squamous cell carcinoma and promotes tumor progression by targeting SOCS2. Biochem Biophys Res Commun.

[20] Re M, Magliulo G, Gioacchini FM, Bajraktari A, Bertini A, Ceka A (2017). Expression Levels and Clinical Significance of miR-21-5p, miR-let-7a, and miR-34c-5p in Laryngeal Squamous Cell Carcinoma. Biomed Res Int.

[21] Bersani C, Mints M, Tertipis N, Haeggblom L, Nasman A, Romanitan M (2018). MicroRNA-155, -185 and -193b as biomarkers in human papillomavirus positive and negative tonsillar and base of tongue squamous cell carcinoma. Oral Oncol.

[22] Hui AB, Lin A, Xu W, Waldron L, Perez-Ordonez B, Weinreb I (2013). Potentially prognostic miRNAs in HPV-associated oropharyngeal carcinoma. Clin Cancer Res.

[23] Li Z, Huang C, Bao C, Chen L, Lin M, Wang X (2015). Exon-intron circular RNAs regulate transcription in the nucleus. Nat Struct Mol Biol.

[24] Ashwal-Fluss R, Meyer M, Pamudurti NR, Ivanov A, Bartok O, Hanan M (2014). circRNA biogenesis competes with pre-mRNA splicing. Mol Cell.

[25] Faustino-Rocha A, Oliveira PA, Pinho-Oliveira J, Teixeira-Guedes C, Soares-Maia R, da Costa RG (2013). Estimation of rat mammary tumor volume using caliper and ultrasonography measurements. Lab Anim (NY).

[26] Bartholomeusz C, Gonzalez-Angulo AM (2012). Targeting the PI3K signaling pathway in cancer therapy. Expert Opin Ther Targets.

[27] Fruman DA, Chiu H, Hopkins BD, Bagrodia S, Cantley LC, Abraham RT (2017). The PI3K Pathway in Human Disease. Cell.

[28] Roux PP, Blenis J (2004). ERK and p38 MAPK-activated protein kinases: a family of protein kinases with diverse biological functions. Microbiol Mol Biol Rev.

[29] Guan G, Zhang D, Zheng Y, Wen L, Yu D, Lu Y (2014). microRNA-423-3p promotes tumor progression via modulation of AdipoR2 in laryngeal carcinoma. Int J Clin Exp Pathol.

[30] Zaman S, Jadid H, Denson AC, Gray JE (2019). Targeting Trop-2 in solid tumors: future prospects. Onco Targets Ther.

[31] Zhang K, Jones L, Lim S, Maher CA, Adkins D, Lewis J (2014). Loss of Trop2 causes ErbB3 activation through a neuregulin-1-dependent mechanism in the mesenchymal subtype of HNSCC. Oncotarget.

[32] Cubas R, Zhang S, Li M, Chen C, Yao Q (2010). Trop2 expression contributes to tumor pathogenesis by activating the ERK MAPK pathway. Mol Cancer.

[33] Cubas R, Li M, Chen C, Yao Q (2009). Trop2: a possible therapeutic target for late stage epithelial carcinomas. Biochim Biophys Acta.

[34] Lydiatt WM, Patel SG, O'Sullivan B, Brandwein MS, Ridge JA, Migliacci JC (2017). Head and Neck cancers-major changes in the American Joint Committee on cancer eighth edition cancer staging manual. CA Cancer J Clin.

[35] Alsbeih G, Al-Harbi N, Bin Judia S, Al-Qahtani W, Khoja H, El-Sebaie M (2019). Prevalence of Human Papillomavirus (HPV) Infection and the Association with Survival in Saudi Patients with Head and Neck Squamous Cell Carcinoma.

[36] Shivdasani RA (2006). MicroRNAs: regulators of gene expression and cell differentiation. Blood.

[37] Arvidsson Y, Rehammar A, Bergstrom A, Andersson E, Altiparmak G, Sward C (2018). miRNA profiling of small intestinal neuroendocrine tumors defines novel molecular subtypes and identifies miR-375 as a biomarker of patient survival. Mod Pathol.

[38] Zhao Y, Lu G, Ke X, Lu X, Wang X, Li H (2016). miR-488 acts as a tumor suppressor gene in gastric cancer. Tumor Biol.

[39] Nakanishi H, Taccioli C, Palatini J, Fernandez-Cymering C, Cui R, Kim T (2014). Loss of miR-125b-1 contributes to head and neck cancer development by dysregulating TACSTD2 and MAPK pathway. Oncogene.

[40] Dong R, Ma XK, Li GW, Yang L (2018). CIRCpedia v2: An Updated Database for Comprehensive Circular RNA Annotation and Expression Comparison. Genomics Proteomics Bioinformatics.

[41] Mitra A, Pfeifer K, Park KS (2018). Circular RNAs and competing endogenous RNA (ceRNA) networks. Transl Cancer Res.

[42] Liu J, Song S, Lin S, Zhang M, Du Y, Zhang D (2019). Circ-SERPINE2 promotes the development of gastric carcinoma by sponging miR-375 and modulating YWHAZ.

[43] Li XN, Wang ZJ, Ye CX, Zhao BC, Huang XX, Yang L (2019). Circular RNA circVAPA is up-regulated and exerts oncogenic properties by sponging miR-101 in colorectal cancer. Biomed Pharmacother.

[44] Han S, Witt RM, Santos TM, Polizzano C, Sabatini BL, Ramesh V (2008). Pam (Protein associated with Myc) functions as an E3 ubiquitin ligase and regulates TSC/mTOR signaling. Cell Signal.

