# Supplementary material for: miR-488-3p sponged by circ-0000495 and mediated upregulation of TROP2 in head and neck squamous cell carcinoma development
Source: J Cancer. 2020 Mar 5;11(11):3375–86. doi: 10.7150/jca.40339 (PMC7097962; doi:10.7150/jca.40339)
Supplement: Supplementary file 1 — Supplementary figure and table. [file jcav11p3375s1.pdf]

Supplementary Table 1

Primers for qRT-PCR

Genes		Primers
circ-0000495	Forward	5'- CATCAGAAGCTGCCCTAACC -3'
	Reverse	5'- TAACTCACCTGGGGGAAAAA -3'
Linear 0000495	Forward	5' -TCATCCATCCCTGTGTCTCA-3'
	Reverse	5' -AAAGAGGCAAGCACAGGAAA-3'
TROP2	Forward	5’-GGACATCAAGGGCGAGTCTCTA-3’
	Reverse	5’-AGGCGCTTCATGGAGAACTTCG-3’
GAPDH	Forward	5' -GTCTCCTCTGACTTCAACAGCG-3'
	Reverse	5' -ACCACCCTGTTGCTGTAGCCAA-3'

Supplementary Figure 1

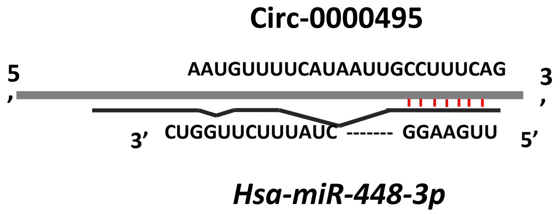

Schema showing circ-0000495 interaction with its target miR-488-3p. The seed sequence was shown in red.
